# A modular analysis of microglia gene expression, insights into the aged phenotype

**DOI:** 10.1186/s12864-019-5549-9

**Published:** 2019-02-28

**Authors:** Christine E. Cho, Sagar S. Damle, Edward V. Wancewicz, Swagatam Mukhopadhyay, Christopher E. Hart, Curt Mazur, Eric E. Swayze, Fredrik Kamme

**Affiliations:** Ionis Pharmaceuticals, Inc, Carlsbad, CA 92010 USA

**Keywords:** Microglia, Transcriptome, Gene modules, WGCNA, Aging, Inflammation

## Abstract

**Background:**

Microglia are multifunctional cells that are key players in brain development and homeostasis. Recent years have seen tremendous growth in our understanding of the role microglia play in neurodegeneration, CNS injury, and developmental disorders. Given that microglia show diverse functional phenotypes, there is a need for more precise tools to characterize microglial states. Here, we experimentally define gene modules as the foundation for describing microglial functional states.

**Results:**

In an effort to develop a comprehensive classification scheme, we profiled transcriptomes of mouse microglia in a stimulus panel with 96 different conditions. Using the transcriptomic data, we generated fine-resolution gene modules that are robustly preserved across datasets. These modules served as the basis for a combinatorial code that we then used to characterize microglial activation under various inflammatory stimulus conditions.

**Conclusions:**

The microglial gene modules described here were robustly preserved, and could be applied to in vivo as well as in vitro conditions to dissociate the signaling pathways that distinguish acutely inflamed microglia from aged microglia. The microglial gene modules presented here are a novel resource for classifying and characterizing microglial states in health and disease.

**Electronic supplementary material:**

The online version of this article (10.1186/s12864-019-5549-9) contains supplementary material, which is available to authorized users.

## Background

1Microglia have relatively recently emerged as important regulators of brain homeostasis, with roles in neuronal survival and proliferation, synaptic pruning, and immune response [1–4]. Microglia actively survey the environment in their basal state, and upon encountering a stimulus change their gene expression and secretory profiles [5].

An increasing appreciation of microglia in health and disease has led to a significant therapeutic interest in microglia and neuroinflammation. Given microglia’s malleable phenotype, characterizing microglia activation states in disease has become a key issue [6]. Our ability to detect and classify microglia activation states is rapidly evolving. Initially, microglia were classified as ‘activated’ or not, mainly based on morphology. The idea of anti-inflammatory and pro-inflammatory states was introduced to the field of microglia in 2006 by Butovsky et al., and the M1/M2 classification originating from macrophages was adopted by subsequent publications [7, 8]. More recently, studies have uncovered states that do not align with the conventional M1/M2 paradigm, implying that a binary classification is insufficient and that there are additional microglia states [9–11]. Therefore, there is a clear need for an improved classification scheme that can adequately define and help describe the molecular basis for microglial phenotypes.

Analysis of co-expression patterns from genome-wide transcriptional profiling datasets provides a powerful means to dissect the molecular basis of cellular behavior and state [12]. For example, a recent study of alveolar macrophages used 28 different treatments to generate 49 gene modules [13]. The modules were then applied to macrophage transcriptomes of healthy and Chronic Obtsructive Pulmonary Disease (COPD) patients, and the authors identified a loss of inflammatory module signatures in the COPD patients that was distinct from the canonical M1 signature. Having a similarly comprehensive resource for microglia would allow us to obtain cell-type-specific modules that form the basis of understanding microglial activation in disease.

In this study, we induced a broad spectrum of activation states in mouse microglia by using a stimulus panel of 96 different treatments. Gene expression changes were organized into 33 modules by Weighted Gene Correlation Network Analysis (WGCNA) [14]. The modules were highly reproducible and had the resolution to distinguish between closely related signaling pathways. We developed a combinatorial code based on the modules, and used it to distinguish between microglia in numerous activation states. Additionally, we identified transcription factors whose known binding sites were enriched within gene modules. In vivo, we used modules to characterize aging, the dominant risk factor for many neurodegenerative diseases. The results of this study will serve as a new resource for classifying microglial activation, and provides a foundation for manipulating microglial phenotypes in disease.

## Results

### A panel of stimuli elucidates microglial gene modules

Microglia can take on a variety of states, characterized by altered gene expression, morphology, and function. In order to induce a diverse array of microglial states, we constructed a stimulus panel comprising 96 different conditions. The panel consisted of 37 unique stimuli including cytokines, pharmacological inhibitors, and molecules known to act in the brain milieu such as ATP and dopamine. Stimuli were applied to microglial samples individually and in combination at 4, 24 and 72 h. A full list of stimulus conditions and number of replicates is available in Additional file 1: Table S1 and Additional file 2: Table S2, respectively.

**Fig. 1:**
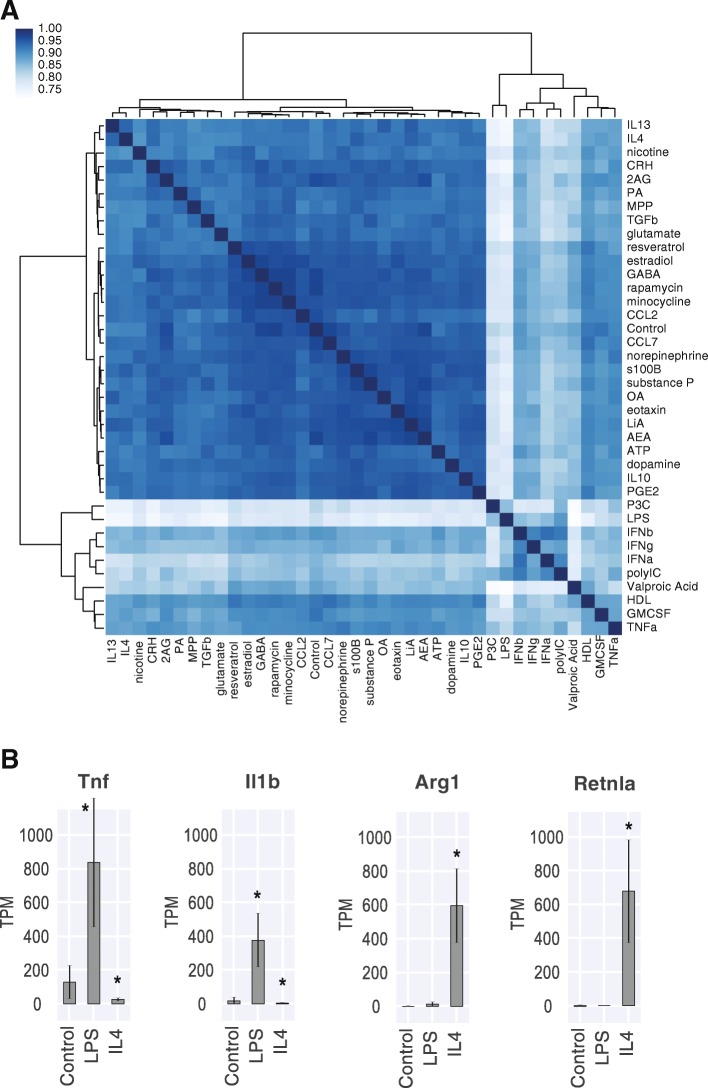
Stimulus panel induces a variety of transcriptomic states in microglia. **a** Hierarchically clustered heatmap of Pearson correlation coefficients between stimulus conditions, based on 6215 most variable genes. **b** Differential expression of canonical markers of LPS (Tnf, Il1b) and IL4 stimulation (Arg1, Retnla). Log2 fold change relative to control. Error bars represent standard deviation. * *p* < 0.05

Following stimulation, 890 samples were profiled by transcriptome sequencing. Of these, 784 samples passed quality control metrics (see Methods). Samples showed high within-condition correlation, confirming the reproducibility between samples (mean Pearson R = 0.937, min = 0.85, max = 0.994). Additionally, we found reduced correlation between many of the known inflammatory stimuli, indicating there was a variety of activation states in our samples (Fig. 1a). Canonical stimuli such as LPS and IL4 showed upregulation of the expected markers (Fig. 1b). There were also a number of stimuli that did not induce a response, such as CCL7, nicotine, and LiA. This result was not surprising, given that our panel included stimuli that have not been shown to affect microglia directly. A full dose titration of each stimulus would be necessary to conclude that microglia are unresponsive to a given stimulus.

**Fig. 2:**
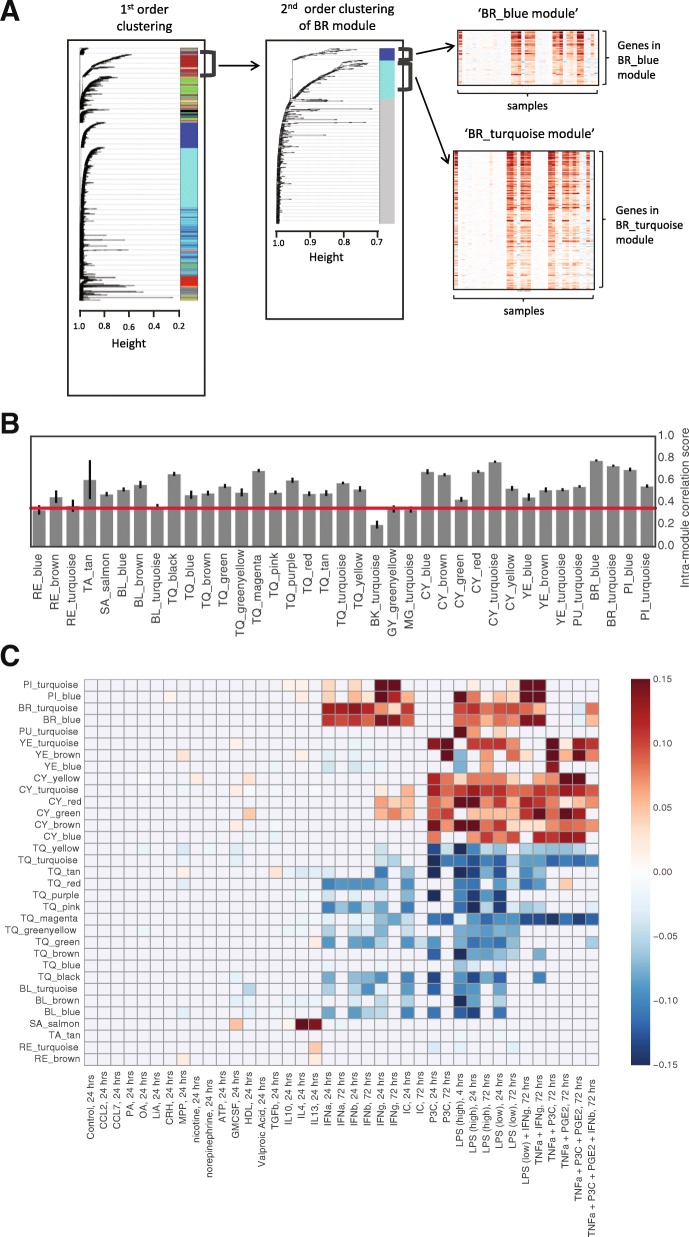
Correlation network analysis reveals microglial gene modules. **a** WGCNA clustering scheme. 1st order clustering was performed on 6215 genes across all core conditions [left]. Each resulting 1st order cluster was then run on WGCNA a second time (2nd order clustering). Example shows 2nd order clustering using the 1st order BR module [center],which yields two 2nd order modules – BR_blue and BR_turquoise. Each 2nd order module comprises a set of co-expressed genes [right]. **b** Mean intramodule correlation score in 37 modules. Red line represents cutoff threshold for reproducibility. Error bars reresent standard deviation. **c** Heatmap of mean module eigengene values across 40 core stimulus conditions. Only modules passing quality control criteria are shown. Heatmap scale is in arbitrary units; a red shade indicates that the module is activated in a given stimulus condition, with darker shades corresponding to stronger activation. Blue indicates suppression of a module. Gray mask = n.s. compared to control, *p < 0.05

In order to identify sets of co-regulated genes, we used WGCNA to cluster 6215 highly-varying genes across stimulus conditions. WGCNA is a robust hierarchical clustering method that employs weighted correlation matrices and adaptive branch cutting to delineate modules of genes that co-vary across samples [14]. The initial clustering step distinguished between the stimulus conditions with the greatest differences, such as IFN and TLR stimuli. We then performed a second clustering step using only the conditions associated with each module (Fig. 2a). This second step allowed us to resolve nuanced gene expression patterns, such as those that distinguish between interferon (IFN) type I and type II stimuli. The two-step clustering method yielded a total of 37 modules, ranging in size from 20 to 148 genes.

We assessed the reproducibility of each module by measuring the intra-module correlation score, which reflects the degree of correlation between the genes that comprise the module (Fig. 2b, Methods). The defining feature of a module is the correlated expression of its constituent genes; as such, only the 33 modules showing high intra-module correlation over 100 bootstrapped datasets were used for downstream analyses. A full list of modules and their member genes can be found in Additional file 3: Table S3.

The activity of a module can be represented by the module eigengene. The eigengene is computed as the first principal component of the genes that make up the module [14], and depicts the dominant trend of expression that is common to those genes. By comparing the module eigengene values across stimulus conditions, one can determine the relative expression of the genes in that module between different conditions. A module was considered active if the module eigengene was differentially expressed between control and test conditions (*p* < 0.05 with Bonferroni correction). Module activity reflects co-regulation of its constituent genes. A comparison of module eigengene activity across conditions showed that all 33 modules have a distinct pattern of regulation (Fig. 2c).

The genes from each module were analyzed by Gene Ontology Enrichment Analysis [15, 16]. Many modules were also associated with GO terms such as “response to cytokine stimulus” and “immune response”, consistent with the known functions of microglia. Furthermore, more specific GO terms were linked to the expected module; for example, “cellular response to interferon-gamma” was exclusively linked to a module that was strongly responsive to interferon gamma (“PI_turquoise”, Fig. 2c). A module responsive to type I interferons (“BR_turquoise”, Fig. 2c) was associated with GO terms such as “response to interferon-beta” and “response to virus”, in line with the known anti-viral role of interferon signaling [17]. Not all modules were associated with a known GO term. This is likely because (1) our modules are microglia-specific whereas gene ontology is derived from many different cell and tissue types, and (2) the resolution of our modules goes beyond the biological pathways that can be found in gene ontology databases. Results for all modules are summarized in Additional file 4: Table S4.

### A combinatorial code of module activation illustrates distinct microglial states induced by IFN I, IFN II, TLR2 signaling

To characterize the transcriptional states in various inflammatory conditions, we compared the transcriptional response to six different stimuli: IFN type I (IFNa and IFNb), IFN type II (IFNg), TLR2 (polyIC), TLR3 (P3C), and TLR4 (LPS).

**Fig. 3:**
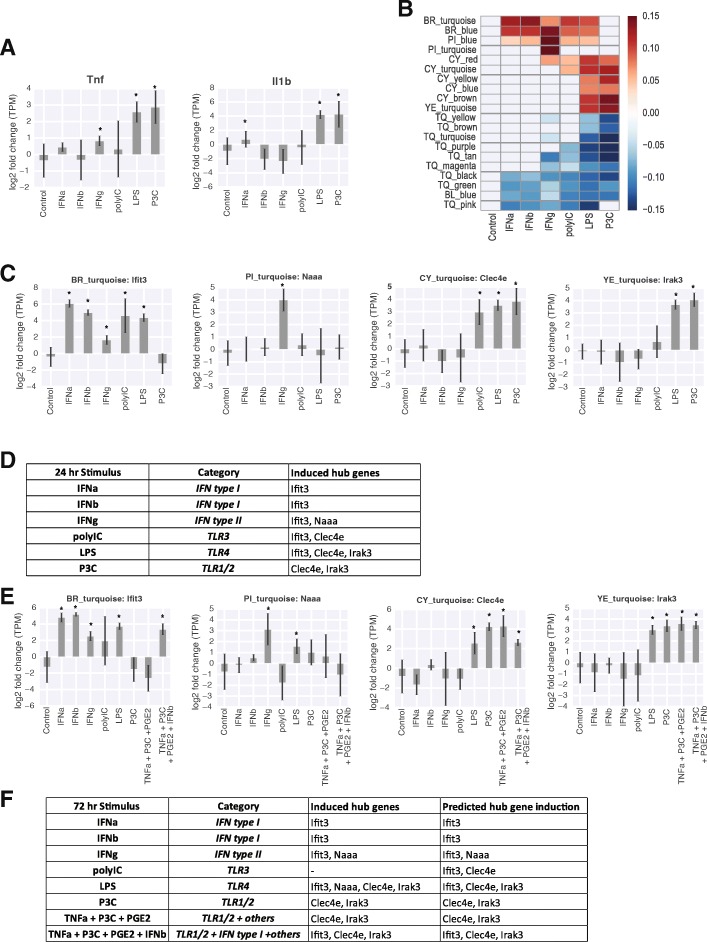
Module markers can be used in a combinatorial code to distinguish between inflammatory states. **a** Differential expression of canonical M1 (Tnf, Il1b) markers upon stimulation with inflammatory stimuli. Log2 fold change relative to control. Error bars represent standard deviation. *p < 0.05. **b** Heatmap of module eigengenes for inflammatory stimuli. Only modules active in at least one condition are shown. Gray mask = n.s. compared to control, *p < 0.05. **c** Differential expression of module markers for BR_turquoise (Ifit3), PI_turquoise (Naaa), CY_turquoise (Clec4e), YE_turquoise (Irak3) modules in 24 h stimulus conditions. Log2 fold change relative to control. Error bars represent standard deviation. *p < 0.05. **d** Combinatorial code of hub gene induction for five different inflammatory stimuli. **e** Differential expression of module markers for BR_turquoise (Ifit3), PI_turquoise (Naaa), CY_turquoise (Clec4e), YE_turquoise (Irak3) modules in 72 h stimulus conditions. Log2 fold change relative to control. Error bars represent standard deviation. *p < 0.05. **f** Comparison of empirical and predicted induction of hub genes

Traditional inflammatory markers such as Tnf and Il1b were upregulated primarily in response to P3C and LPS (Fig. 3a), but were not sensitive to other stimuli associated with neuroinflammation, such as IFNa or IFNb. In contrast, our modules captured responses from all six stimuli, with a given stimulus regulating anywhere from 7 to 19 modules (IFN type I and LPS, respectively) (Fig. 3b). Several modules showed a distinction between IFN I vs II stimuli (BR_turquoise, PI_blue, PI_turquoise), while other modules (CY_yellow, CY_blue, CY_brown, YE_turquoise and TQ_brown) were only regulated by TLR 1/2 and 4 stimuli. Most modules showed regulation in two or more conditions, but notably, no module was upregulated by all six stimuli.

The overlapping patterns of activity could be described by a combinatorial code. Comparing activation of just four modules, such as BR_turquoise, PI_turquoise, CY_turquoise, YE_turquoise, was sufficient to distinguish between the five types of stimuli (Fig. 3b). Note, the two IFN type I stimuli did not show significant difference in any module.

To extend the applicability of these modules to future experiments, we identified hub genes to be used as markers for each module. In this context, hub genes are defined as the genes that show high correlation to the module eigengene and can thus serve as biological markers of module activity (see Methods for hub gene ranking and selection). Figure 3c illustrates how the expression of the hub gene for each of the four modules, BR_turquoise, PI_turquoise, CY_turquoise, YE_turquoise, can be used as a combinatorial code to identify which of the pro-inflammatory stimuli microglia had been exposed to. Figure 3d tabulates the results of Fig. 3c to demonstrate how different signaling pathways are associated with a specific combination of hub genes.

We challenged the robustness of our hub genes using samples from several test conditions. The hub genes were derived from conditions where the stimuli were applied for 24 h. As our test case, we selected conditions where the same stimuli were applied for 72 h, as well as an additional combinatorial stimulus condition (Fig. 3e). Figure 3f compares the actual results to the results that would be predicted based on the code established in Fig. 3d. Nearly all conditions met their prediction; as expected, type I interferons (IFNa and IFNb) could be identified by the induction of Ifit3 and absence of Naaa, while type II interferon (IFNg) induced both Ifit3 and Naaa. Furthermore, combinatorial stimuli that include P3C and IFNb induced signatures for both stimuli. There was a discrepancy in LPS, in that Naaa was induced when it was not predicted based on the original combinatorial code. This is likely due to the amplification of downstream signaling cascades in the 72 h LPS stimulation condition; LPS is known to induce interferon-gamma production in macrophages, and the induction of Naaa is consistent with the presence of interferon gamma [18].

### Genes in IFN-associated modules are selectively down-regulated by resveratrol

We further probed the modular transcriptional activity by examining the effect of anti-inflammatory agents on gene expression. We treated microglia for 24 h with LPS alone or in combination with resveratrol, a natural phenol, or rapamycin, a small-molecule inhibitor of the mTOR pathway.

**Fig. 4:**
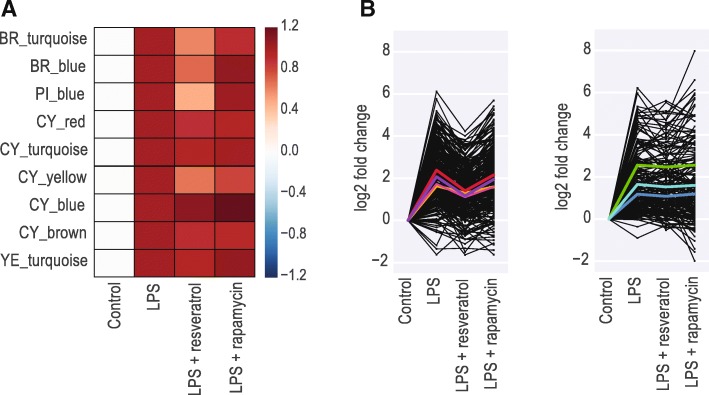
A subset of LPS-induced modules are regulated by resveratrol. **a** Heatmap of median differential expression of module genes, normalized to LPS response. Only modules activated by LPS are shown. **b** Line plots comparing differential expression of genes in modules strongly regulated by resveratrol [left], or unaffected by resveratrol [right]. Each black line represents a single gene. Thick lines represent mean differential expression for each module [red line: BR_blue module, orange line: CY_yellow module, magenta line: PI_blue module, purple line: BR_turquoise, green line: CY_blue module, turquoise line: CY_turquoise module, blue line: YE_turquose module]

Nine modules were activated by LPS alone. Combining rapamycin with LPS did not reduce activity in any of the nine LPS-responsive modules. In contrast, combining resveratrol with LPS reduced gene expression by over 50% in several modules. Notably, this reduction in activity by resveratrol was restricted to some modules, as several other modules still showed the same level of activity as when treated with LPS alone (Fig. 4a). Median decrease in gene expression in the four LPS-responsive modules with greatest effects of resveratrol (BR_blue, BR_turquoise, PI_blue, CY_yellow) was 1.74-fold (Fig. 4b, left). In comparison, there was no change in the LPS-response in other modules such as CY_blue, CY_turquoise, CY_brown (Fig. 4b, right). These results demonstrate that the modules can be independently regulated, and likely represent genes in distinct biological pathways.

We observed strong overlap between the modules regulated by resveratrol and those regulated by interferon stimuli in Fig. 3b indicating potential suppression of the IFN mediated pathways by resveratrol. Gene ontology (GO) enrichment analysis confirmed that the subset of genes regulated by resveratrol is enriched for genes involved in interferon signaling (Table 1). Taken together, this shows that co-stimulation with LPS and resveratrol results in an intermediate activation state in which interferon signaling is ameliorated but other inflammatory signals remain high.

**Table 1:** GO terms associated with genes changed by resveratrol

GO biological process complete	Fold-enrichment	*P*-value
antigen processing and presentation of endogenous peptide antigen (GO:0002483)	48.94	6.92E-04
cellular response to interferon-beta (GO:0035458)	46.37	1.89E-20
response to interferon-beta (GO:0035456)	45.68	3.50E-24
cellular response to interferon-alpha (GO:0035457)	43.51	2.29E-02
response to interferon-alpha (GO:0035455)	41.22	3.17E-07
regulation of response to interferon-gamma (GO:0060330)	40.79	1.69E-03
antigen processing and presentation of endogenous antigen (GO:0019883)	40.79	1.69E-03
cellular response to exogenous dsRNA (GO:0071360)	32.63	5.04E-03
negative regulation of viral genome replication (GO:0045071)	30.12	1.49E-10
positive regulation of interferon-alpha production (GO:0032727)	29.37	6.76E-04
regulation of interferon-alpha production (GO:0032647)	27.41	8.74E-05
cytokine metabolic process (GO:0042107)	25.76	1.59E-02
positive regulation of response to cytokine stimulus (GO:0060760)	25.76	1.06E-07

### Regulatory factors associated with inflammatory modules

**Fig. 5:**
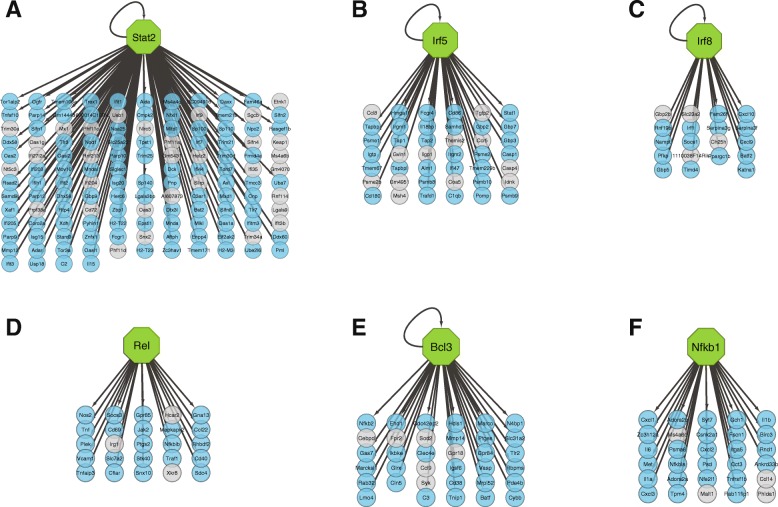
Candidate transcription factors (TF) strongly associated with a module. Green hexagon: TF. Blue circles: module genes with predicted regulation by TF. Gray circles: module genes not predicted to be regulated by TF. **a** BR_turquoise module, (**b**) BR_blue module, (**c**) PI_blue module, (**d**) CY_red module, (**e**) CY_turquoise module, (**f**) CY_brown module

One mechanism by which genes are co-regulated is via control by a common transcription factor. We used iRegulon [19] to identify transcription factors that may be acting as regulators of our gene modules. All modules showed strong association (Normalized Enrichment Score (NES) > 3 as defined by Janky et al., 2014) with at least one transcription factor. Six of the modules, BR_turquoise, BR_blue, PI_blue, CY_red, CY_turquoise and CY_brown, had transcription factors with particularly high NES (> 7), and these were predicted to regulate 72–87% of the genes in the module (Fig. 5, Table 2). The remaining three modules had associated transcription factors with 7 > NES > 5, and the transcription factors were predicted to regulate 32–59% of the genes in their respective modules. Notably, there was little overlap in transcription factors between the modules. This segregation presents a potential biological mechanism for the modular nature of gene expression observed in our data.

**Table 2:** Top transcription factors predicted for each module

Module	TF	NES	predicted regulation (%)
BR_turquoise	Irf9	20.4	73
	Stat2	19.9	76
	Irf7	9.5	52
BR_blue	Irf5	16.4	72
PI_blue	Irf8	12.7	80
	Sp100	7.2	40
	Irf1	6.8	55
	Alx1	6.7	55
CY_red	Relb	7.3	80
	Rel	7	88
	Nr1h3	5.1	32
CY_turquoise	Bcl3	8.6	83
	Irf2	6.5	50
	Sfpi1	6.2	72
CY_yellow	Sox10	5.6	59
CY_blue	Srf	5.6	53
	Irf4	5.2	38
CY_brown	Nfkb1	10	87
	Sox9	5.1	17
YE_turquoise	Atf4	5.3	12
	Ets2	5.2	32

### Modules are regulated in vivo

Studies comparing acutely isolated microglia to microglia in culture have shown that there are a number of transcriptional changes induced by the environment [20]. We wanted to see whether this would affect the modular nature of microglial gene expression, or if the same gene modules could be extended to an in vivo context. Mice were injected i.p. with LPS or vehicle, followed by isolation of microglia for transcriptional profiling at 4 h post injection. Consistent with the literature, comparison of the vehicle-treated in vivo samples to the untreated controls in our in vitro samples showed that there was induction of genes such as Gpnmb, Spp1, and Msr1, and downregulation of genes such as Tmem119, Olfml3, and Sall1 (data not shown).

**Fig. 6:**
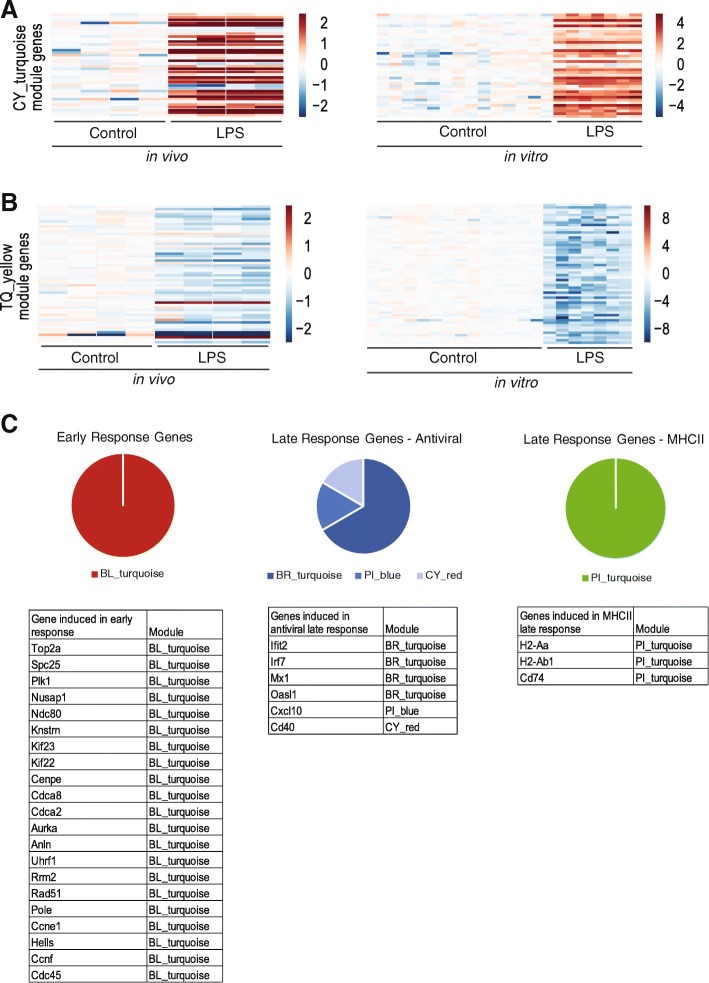
Modules derived in vitro can be observed in vivo (**a-b**) Representative modules upregulated [A] and downregulated [B] by LPS treatment in vivo and in vitro. Heatmaps show of differential expression for the genes in each module (log2 fold change relative to mean expression of control samples). n > =4 samples per condition. **c** Module membership of genes from Mathys et al., (2018) that correspond to the early-response microglia [left], late-response-interferon microglia [middle], and late-response-MHCII microglia [right]. Pie chart [top] shows the proportion of genes from each list corresponding to a given module. Tables [bottom] show the list of genes and their module membership

If a module is preserved in vivo, we would expect the genes to show correlated expression patterns across the in vivo samples, just as we saw in the in vitro samples. It is worth noting that only 15 of the 33 modules could be assessed for preservation, because the remaining modules did not show expression changes in response to LPS in vivo and most likely require a different stimulus. Of the 15 active modules, four modules showed module breakdown, where the module genes did not show correlated expression patterns. The remaining 11 modules were determined to be reproducible, that is, genes within these modules showed correlated expression changes in response to LPS in vivo*.* This indicates that, despite differences in gene expression at baseline, the modular architecture of gene expression was intact (Fig. 6a-b).

We expect true biological modules to be preserved even at the single-cell level. To test whether our modules could translate to single-cell microglial transcriptomes, we used a recent published dataset; Mathys et al., (2018). sequenced individual microglia from CK-p25 mice, an Alzheimer’s disease model with a rapidly progressing neurodegeneration phenotype, and identified subsets of microglia associated with the various stages of neurodegeneration [21]. They found distinct sets of genes upregulated in microglia at different stages of disease. We overlaid the gene sets from Mathys et al., with our modules to see whether their gene sets could be partitioned based on our modules. Figure 6c shows that genes upregulated in microglia in early-stage disease fall within a single one of our modules. Mathys et al., identified two different subsets of late-stage microglia, and these were characterized by BR_turquoise and PI_turquoise modules, respectively (Fig. 6c). Thus, we find that our modules are preserved even at the single-cell level.

### Microglia have distinct activation signatures in acute inflammation and aging

**Fig. 7:**
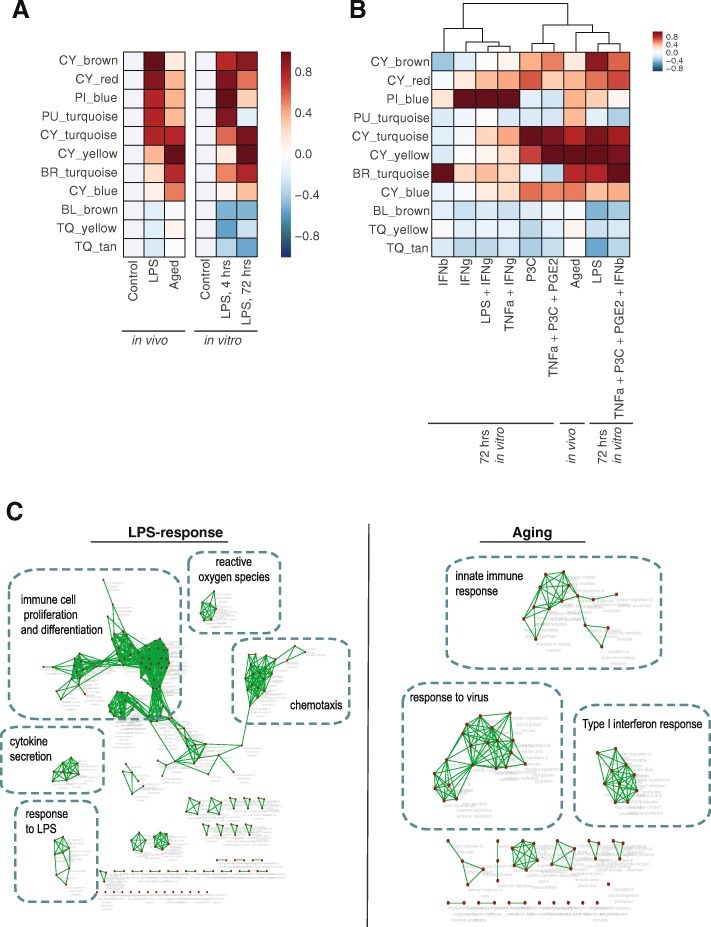
Modules distinguish between acute inflammation and aging in vivo. **a** Heatmap of median differential expression of module genes comparing acute and chronic conditions in vitro and in vivo. Values are normalized by condition. **b** Hierarchical clustering of median differential expression value of modules showing similarities between the in vivo aged condition and eight in vitro chronic stimulation conditions. Values are normalized by condition. **c** Enrichment maps for pathways regulated by LPS treatment [left] or aging [right]

Aging induces a primed phenotype in microglia [22], which is thought to be associated with chronic activation. We isolated microglia from 22-month old mice and compared their gene expression to that of the LPS-treated mice. Comparison of the most active modules in the two conditions revealed a differential response; LPS treatment most strongly involved modules CY_brown, CY_red, PU_turquoise, PI_blue, and CY_turquoise, whereas in aging, the primary modules were CY_yellow, BR_turquoise, and CY_turquoise (Fig. 7a, left). Notably, this differential activation was also observed in vitro with acute (4 h) or chronic (72 h) LPS treatment respectively (Fig. 7a, right). Upon prolonged exposure to LPS in vitro, the microglial transcriptional response shifted from stronger activation of CY_red, PI_blue, and PI_brown modules to activation of BR_turquoise, CY_turquoise, and CY_yellow modules. To determine whether other chronic stimuli could also model the aged phenotype, we examined module activation in seven additional chronic stimulation conditions. Heirarchical clustering shows that, with the exception of LPS, no individual stimulus induced all three of the top aging-associated modules. However, we observed that of these three modules – CY_yellow, CY_turquoise, and BR_turquoise – two are strongly induced by chronic P3C stimulation and one is strongly induced by chronic IFN type I stimulation, indicating that each may contribute to part of the aging phenotype. Indeed, when P3C and IFNb were both included in a combined stimulus, this more closely recapitulated the aged phenotype (Fig. 7b).

**Fig. 8:**
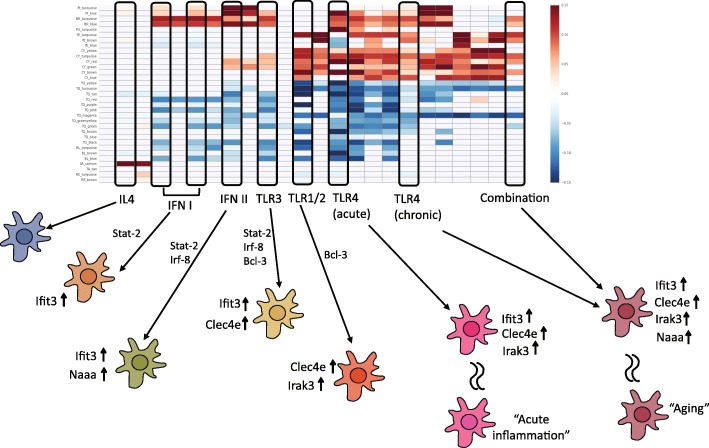
Gene modules distinguish between many different microglial states. Module activation patterns characterize microglia in various states. Hub gene markers of each modules can be used in a combinatorial code that distinguishes between states. The constituent genes from each module were used to predict transcription factors that may regulate each state

Pathway analysis revealed the biological processes associated with the top modules activated in each condition (Fig. 7c). Modules activated in microglia from LPS treated animals were enriched for pathways related to proliferation, chemotaxis, reactive oxygen species production, and cytokine production. In contrast, modules in aging microglia were enriched for IFNa and IFNb signaling, and response to viral infection. Our analysis demonstrates the utility of gene expression modules to robustly detect different activation states of microglia in vivo, in this case acute inflammation and aging. As modules correspond to known stimuli and defined transcriptional activators, differences in module activation provide information on signaling involved in each microglia activation state (Fig. 8). Finally, we show that some aspects of aged microglia in vivo can be recapitulated during chronic stimulation in vitro.

## Discussion

Here we present a framework for understanding microglial states using gene expression modules. Using 96 stimulus conditions, we induced a wide variety of microglial transcriptional programs and categorized a complex transcriptional response into concise gene expression modules using a clustering-based approach. We demonstrate the utility of these modules in identifying numerous microglial activation states.

Modular organization of gene expression has been shown in other organisms and cell types [23–25]. Previous studies have identified co-expression modules in microglia associated with aging, as well as different brain regions [11, 26]. While these works provide us with a useful way of understanding gene expression changes in specific contexts, the small number of conditions in these studies limits the level of the detail the modules can provide; typically, these modules can only be associated with the age or disease status included in the experiment. In contrast, we designed our stimulus panel with the intention of isolating modules that are capable of resolving individual signaling pathways implicated in a broad spectrum of microglia-related pathologies. We successfully identified 33 modules that distinguish between closely related stimuli.

A small number of modules showed co-regulation in vitro but not in vivo, which may be attributed to the transcriptional changes that take place when microglia are removed from the CNS environment. A greater number of modules showed the same co-regulation in vivo as they did in vitro, indicating that despite transcriptional changes at baseline, the network connectivity between these genes is unaffected. The fact that a subset of these modules could be observed in an independent single-cell dataset supports the idea that these modules can be applied in vivo.

Using gene modules provides several advantages in describing microglial state. The use of single marker genes such as Tnf and Arg1, while indicative of general activation, often cannot resolve different inflammatory states, such as IFN-activated versus LPS-activated microglia. We propose a combinatorial code of gene module activity to describe microglial states. As demonstrated, a combinatorial module code robustly differentiated between type I IFNs, IFNg, PolyIC, LPS and P3C in vitro, and acute LPS-induced inflammation and chronic aged-induced inflammation in vivo (Fig. 8).

A descriptive system based on modules carries a significant practical benefit: hub genes can be used to report on the activity of their respective modules. Hub genes offer the same tractability as the current individual marker genes, but are representative of a defined set of co-regulated genes and associated biological pathways. Hub gene expression can thus be used, in lieu of complete transcriptome sequencing, to characterize microglia activation states. As an example, we show that six pro-inflammatory conditions in vitro can be distinguished using four hub gene markers (Fig. 3c). For in vivo work, this also opens up the possibility of using multiplexed in situ hybridization to characterize microglia activation states.

In addition to addressing the issue of characterizing microglial activation states, our results give novel insight into inflammatory signaling pathways. Although it has long been known that IFN type I and type II signal through different receptors [27], the effect of each on microglial gene expression was not fully known. We find there is a group of genes commonly induced by both types of interferons, which include Stat1 and Cd86. We also find groups of genes that are preferentially induced by type I or type II interferon signaling. Genes such as Ifit1, C2, Lgals3bp and Irf7 were associated with type I signaling, whereas genes such as Cxcl9 and Socs1 were more strongly induced by type II signaling. Notably, the group of genes including Naaa, Zyx and Clec9a were highly specific to interferon type II response and was not induced by any other stimulus tested.

These gene modules also provide a strong basis to uncover regulators that drive microglial states. The transcription factors associated with each module present a potential biological mechanism by which the modular regulation may be achieved. Interestingly, several of these, such as Irf8 and Nfkb1, have been previously identified as Signal Dependent Transcription Factors that may regulate microglial phenotype in neuropathic pain and neurodegeneration, respectively [28, 29]. It will be interesting to see whether the microglia in these disease scenarios correspond to upregulation of the modules found in our study. Additionally, the transcription factors identified here could be tested for use as therapeutic targets to downregulate modules of interest. For modules that did not show strong association with any particular transcription factor, microRNAs or other factors may be acting to further regulate subsets of genes. Future studies should examine potential regulatory regions shared by genes in a given module.

Chronic activation phenotypes are of particular relevance to the pathological role of microglia. Indeed, this is a central consideration when interpreting the role of microglia in various neurological conditions that become more prevalent with age, such as Alzheimer’s disease and Amyotrophic Lateral Sclerosis [30–33]. Here, we profiled microglia from aged wild-type mice to study the microglial priming phenotype. The gene expression pattern in aged mice corresponded to the up-regulation of three gene modules, BR_turquoise, CY_turquoise and CY_yellow. Our stimulus panel shows that the BR_turquoise module was activated by IFN type I stimuli (Fig. 3), in line with results from previous studies that have shown upregulation of IFN type I signaling with aging [34]. The other two modules, CY_turquoise and CY_yellow, were activated by the TLR3 stimulus in our panel (Fig. 3). Furthermore, pathway analysis results comparing the BR_turquoise module and the CY_turquose + CY_yellow module show that former is responsible for activating the viral response, IFN-alpha and IFN-beta pathways, whereas the latter is associated with innate immune response and migration (Fig. 7c). In effect, we have successfully dissected the microglial aging signature further into two separate components; one, a known interferon type I signal, and the other, a previously uncharacterized gene signature induced by TLR3 signaling. This analysis provides testable hypotheses for reversing age-induced molecular changes in microglia.

Comparison of aging and acute inflammation in vivo revealed that distinct sets of modules dominate the microglial response in these two perturbations (Fig. 7a, left). Pathway analysis showed upregulation of proliferation and migration during acute inflammation, which was not seen in aged microglia, pointing to different functional properties in acutely vs chronically inflammatory microglia. This is an important point from a therapeutic viewpoint – anti-inflammatory drugs targeting canonical inflammatory cascades may not counteract the chronic inflammation seen in aged microglia.

We were able to recapitulate portions of the aging-associated module activation pattern in several of our in vitro conditions. Comparison of 4- and 72-h LPS stimulation showed a similar distinction to that of acute LPS-treatment and aging in vivo, respectively (Fig. 7a, right). In the case of 72-h LPS stimulation in vitro, we found induction of one additional module, CY_brown, that was not prominent in aging in vivo. This module may be unique to LPS, or may reflect the limitations of recapitulating the aged phenotype in vitro. Despite this difference, our results indicate that aged microglia may be modeled in part by chronic LPS stimulation in vitro, providing a useful way to model certain aspects of microglial priming in a short time span. Additionally, we observed that chronic stimulation with IFNb or P3C induced non-overlapping subsets of the aging-associated modules, suggesting that interferon type I and TLR1/2 signaling could both contribute to the aging phenotype. Future experiments should address which of these modules can be associated with microglial dysfunction.

Microglia stimulated by LPS are known to release a plethora of cytokines that in turn activate other receptors besides TLR4 [35–37]. As such, gene expression changes in our 72-h stimulus conditions may represent a response to endogenous signals released by microglia, rather than a response to the exogenous stimulus itself. This raises the possibility that the aged phenotype of microglia in vivo is similarly a response to autonomous signaling in response to chronic activation.

## Conclusions

This work provides a comprehensive dataset that can be used as the basis for classifying microglial phenotypes. First and foremost, we find that microglial gene expression is modular in nature, and the gene modules derived here can be used to characterize microglial states both in vitro and in vivo*.* The combinatorial code of module activity can distinguish between states induced by a variety of stimulus types and treatment durations; distinct transcriptional activation states were induced by IFN type I, IFN type II, TLR2, TLR3 and TLR4 signaling. The in vitro stimulus panel itself supplements traditional pathway and regulator analysis, as it is a microglia-specific resource with experimentally validated annotations that link gene co-expression to stimulus. Utilizing this feature, we show that aging microglia are in an activation state that is dominated by IFN type I and TLR3 signals, providing new insight for targeting senescence of the neuro-immune system. In summary, our data provide a novel resource for elucidating microglial states.

## Methods

### Primary cell culture

Primary neonatal mouse microglia (Sciencell, Cat#M1900–57) were cultured in poly-lysine coated 96-well plates with Microglia Medium (Sciencell, Cat #1901). Cultures were kept in an incubator at 37 °C, 5% CO_2_. Media was refreshed 24 h after plating. 4 days after plating, media was removed and replaced with fresh media containing a stimulus.

### Animals

Animal studies were conducted under a protocol approved by the Ionis Institutional Animal Care and Use Committee (IACUC) in an Association for Assessment and Accreditation of Laboratory Animal Care (AALAC) approved animal facility. For the LPS-stimulation experiment, female C57BL/6 mice (Jackson Laboratories), 2 month old, were injected intraperitoneally with 2 mg/kg LPS (Sigma, strain 0111:B4) formulated at 0.5 mg/ml in PBS (*n* = 4). At 4 h post dosing, microglia were isolated as described below. For comparison of aged and young mice, we isolated microglia from male C57BL/6 mice at 22-month and 2-months of age respectively (*n* = 12).

### Microglial isolation

Mice were deeply anesthetized by isoflurane inhalation (4% in air carrier gas) in an induction box and maintained on a deep plane of anesthesia using a nose cone. The anesthetized animals were perfused with ice-cold PBS and whole brains were collected in 1X HBSS+HEPES buffer. Brains were finely minced with a razorblade and digested in Accutase (EMD Millipore, Cat #SCR005) at 4 °C for 45 min. Following successive trituration with clipped pipette tips of decreasing diameter, cells were washed with 1X HBSS+HEPES and filtered with a 250 uM mesh filter. Tissue homogenates were centrifuged over a 30% Percoll gradient to remove myelin. Microglia were isolated by Magnet Activated Cell Sorting (MACS) using a CD11b antibody (Miltenyi).

While Cd11b is expressed on peripheral monocytes in addition to microglia, our method of perfusion removes blood contamination from the CNS and thus minimizes peripheral monocyte contamination of our sample from peripheral monocytes. We confirmed the purity of this method using flow cytometry. Previous studies have shown that flow cytometry analysis of CD45 levels distinguishes between CD11b-positive microglia and macrophages, with microglia expressing CD45 at low levels and macrophages expressing it at high levels [38]. Our flow cytometry analysis shows that 97.3% of the cells in our CD11b-isolated microglial samples are CD45-low, and only 1.4% are CD45-high (Additional file 5: Figure S1).

Furthermore, we examined cell-type specific marker expression of the microglial samples used in our analyses. Our samples show high expression of the microglia-specific markers, while having little to no expression for markers of other immune cell types (Additional file 6: Figure S2). The markers for each cell-type were derived by single-cell analyses from brain immune cell populations [39], which have none of the confounding effects of contaminated populations, as well as other seminal papers that have focused on differentiating microglia from other CNS immune cell types [40, 41].

### RNA extraction, library preparation

RNA extraction was performed using Qiagen RNeasy kits. 0.5–1 ng of total RNA was used as template for the initial reverse transcription, which included 0.27 uM of barcoded primer, containing a T7 promoter sequence. 48–96 cDNA samples were pooled, followed by a 8 h T7 amplification. 50 ng of the resulting aRNA was used as input for library generation using the Quantseq aRNA kit (Lexogen, Cat #043.24.V0.1).

### Next generation sequencing

NGS libraries were sequenced as 75 bp fragments with a median depth of 6.1 million reads per sample on an Illumina NextSeq500. Transcript quantitation was performed with Salmon (ver 0.7.1) using quasi-mapping based mode with automated libtype detection [42]. Gene level TPM was computed by summing read counts of all associated transcript isoforms and normalizing by total number of mapped reads. Gene model indexes were generated from Ensembl *Mus musculus* build 81 [43]. The median alignment rate through salmon was 81.5%. Samples with less than 1 million mapped reads or less than 6500 genes expressed (TPM > 5) were discarded. Differential gene expression was assessed by comparing against a negative binomial error model based on gene expression in control microglia samples. Gene-specific *p*-values were computed for each biological replicate and median-aggregated. Genes having a minimum p-value less than or equal to 0.05 in all replicates within a group were considered significant and used in downstream analysis.

### Weighted gene correlation network analysis (WGCNA) analysis

A group of 6215 genes were selected based on top 4000 genes that were highly variable across all conditions and an additional 2215 genes that were differentially expressed in at least one condition. Of the 96 conditions tested, we selected 40 core conditions as input to the WGCNA analysis. Many of the 96 conditions consisted of LPS combined with a potential modulator (ie., rapamycin), and we excluded these from the input. As the WGCNA method relies on variability between samples, the inclusion of all LPS + modulator conditions would drive WGCNA to primarily detect the LPS response and lose sensitivity to more subtle responses from other stimulus conditions. Hence, our input conditions were designed to prevent biasing WGCNA results towards the LPS response. Each condition had a minimum of four replications from two different experimental days, for a total of 373 samples. It is well known that gene expression is distributed as a Negative Binomial distribution -genes with a higher expression level typically exhibit higher variability in expression than expected from Poisson behavior [44]. Clustering algorithms assume Gaussian noise around cluster centers and underlying distance measures reflect this assumption. In order to mitigate the dependence of variance on mean expression we transformed the expression level by using the function for Negative Binomial distribution, such that the transformed variable is Gaussian distributed -a process known as variance stabilization [45]. The function for Negative Binomial distribution, and this stabilized expression matrix was used as input for WGCNA (ver 1.51).

For the first round of clustering, the adjacency matrix was calculated with a soft power of 4. The soft power was selected based on the standard connectivity analysis for WGCNA; briefly, we chose the lowest soft power value where the scale-free topology threshold is met [14]. The cutreeDynamic function was run with the following parameters: deepSplit = 1, PAMstage = True, minClusterSize = 20, method = hybrid. The mergeCloseModules function and was run with a cut height of 0.25. The resulting 14 modules were clustered in a second round, using matrices consisting of the genes in a given first order module and the samples associated with the module. The cutreeDynamic parameters for the second-round clustering were as follows: deepSplit = 4, PAMstage = False, minClusterSize = 20, method = hybrid. Three of the first order modules remained as a single module after the second round of clustering, indicating that additional modules were not forced in cases where the 1st order cluster was already optimal.

### Module quality analyses

Module reproducibility was determined by the intra-module correlation score. We derived the score by first ranking all genes in a given module according to their mean Pearson correlation coefficient against other genes in the module. The correlation coefficient of the gene at the 75th percentile was then selected as the intra-module correlation score. We repeated the measure on 100 independent subsets of the data, which were obtained by randomly selecting half of the samples in each condition. The resulting scores were used to compute mean and variance. Modules with a mean score greater than 0.35 were considered reproducible.

The in vivo status of modules was assessed by quantifying activation and reproducibility. Activation was defined as a 1.5-fold change in gene expression compared to control. Modules in which less than a third of the genes did not meet the activation threshold were categorized as inactive, and these modules excluded from further analysis. Reproducibility was defined as an intra-module correlation score greater than 0.35 based on the active genes in a given module.

### Hub gene selection

Genes in each module were first ranked by correlation to the module eigengene*—*the module eigenegene is defined as the first principle component in the gene space of a given module [14]. Of the ten genes with the highest correlation, the gene with the lowest coefficient of variance and minimum expression of at least 5 TPM was selected as the hub gene for the module.

### Network and pathway analysis

We used the web-based application gProfiler [46] to obtain significantly enriched GO biological process and Reactome pathways, excluding electronic GO annotations. The resulting pathways were then displayed using the Enrichment Map app in Cytoscape 3.0 using Jaccard similarity coefficient with an FDR cutoff of 0.0001 [47, 48].

For transcription factor prediction, we used the cytoscape plugin GeneMANIA to first create networks between the genes in each module [49]. The network was analyzed using the cytoscape plugin iRegulon (‘Predict regulators and targets’ function, motif collection = 10 K (9213 PWMs)) to rank motifs around the 20 kb centered around the transcriptional start site. TFs were predicted with maximum FDR of 0.001 in motif similarity.

### Statistical analysis

Mann-Whitney U tests were performed using the Python scipy package. *p* < 0.05 after Bonferroni correction was considered significant.

## Additional files

Additional file 1: **Table S1** List of conditions used in microglia stimulus panel. Table containing stimulus identity, supplier, stimulus duration and concentration for all conditions used in the stimulus panel. (CSV 3 kb)

Additional file 2: **Table S2** Number of replicates in each stimulus condition. Table showing the number of biological replicates in each condition. (CSV 2 kb)

Additional file 3: **Table S3** Lists of genes in each module. Each column shows the full list of genes in a given module. (CSV 13 kb)

Additional file 4: **Table S4** Gene Ontology (GO) terms associated with each module. Columns show the GO term and corresponding q-value associated with each module. Some modules did not yield any significant GO terms. (CSV 9 kb)

Additional file 5: **Figure S1** Flow cytometry shows enrichment of CD45low cells in Cd11b-MACS samples. (A) Flow cytometry of Cd45 in a representative Cd11b-MACS sample [left] and a positive control containing all CNS immune cell types [right]. (PDF 426 kb)

Additional file 6: **Figure S2** Cd11b-MACS samples express microglia-specific markers. (A) Expression of various in immune cell markers in MACS-Cd11b samples. Error bars represent standard deviation. (B) Table listing the cell type associated with each marker gene. (PDF 493 kb)

## Abbreviations

AAALAC: Association for Assessment and Accreditation of Laboratory Animal Care
CNS: Central Nervous System
COPD: Chronic Obstructive Pulmonary Disease
FDR: False Discovery Rate
GO: Gene Ontology
IACUC: Institutional Animal Care and Use Committee
IFN: Interferon
LiA: Linoleic Acid
LPS: Lipopolysaccharide
MACS: Magnet Activated Cell Sorting
NES: Normalized Enrichment Score
P3C: Pam3CysSerLys4
polyIC: Polyinosinic-polycytidylic acid
PWM: Position Weight Matrix
TLR: Toll-like Receptor
WGCNA: Weighted Gene Correlation Network Analysis
