# Supplementary figures and images for: A modular analysis of microglia gene expression, insights into the aged phenotype
Source: BMC Genomics. 2019 Feb 28;20:164. doi: 10.1186/s12864-019-5549-9 (PMC6396472; doi:10.1186/s12864-019-5549-9)

Fig. S1

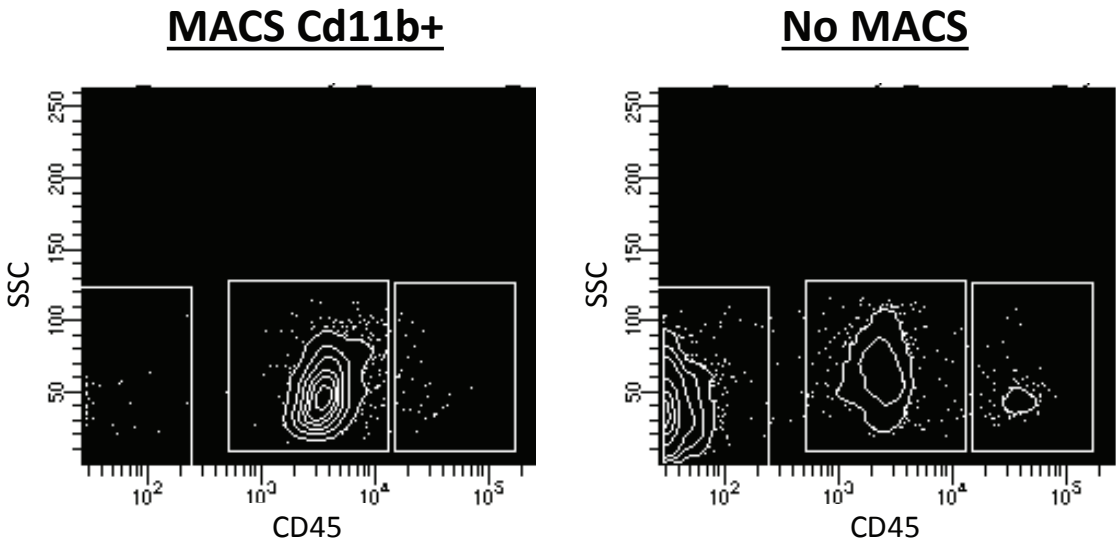

Supplement: Supplementary file 5 — Figure S1 Flow cytometry shows enrichment of CD45low cells in Cd11b-MACS samples. (A) Flow cytometry of Cd45 in a representative Cd11b-MACS sample [left] and a positive control containing all CNS immune cell types [right]. (PDF 426 kb) [file 12864_2019_5549_MOESM5_ESM.pdf]
