# Supplementary material for: A modular analysis of microglia gene expression, insights into the aged phenotype
Source: BMC Genomics. 2019 Feb 28;20:164. doi: 10.1186/s12864-019-5549-9 (PMC6396472; doi:10.1186/s12864-019-5549-9)
Supplement: Supplementary file 6 — Figure S2 Cd11b-MACS samples express microglia-specific markers. (A) Expression of various in immune cell markers in MACS-Cd11b samples. Error bars represent standard deviation. (B) Table listing the cell type associated with each marker gene. (PDF 493 kb) [file 12864_2019_5549_MOESM6_ESM.pdf]

Fig. S2

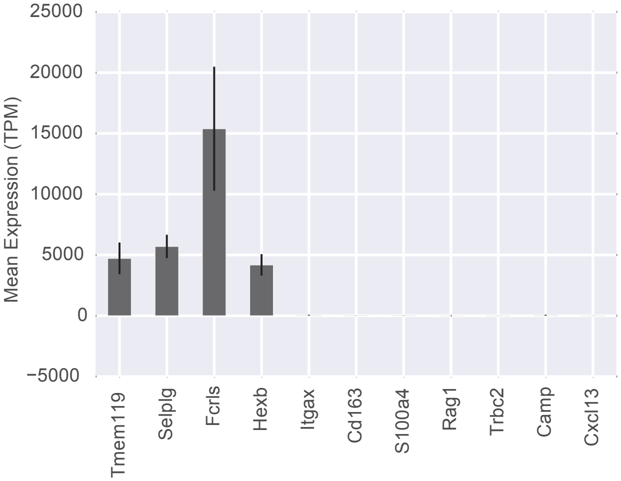

Gene	Cell Type
Tmem119	Microglia
Selplg	Microglia
Fcrls	Microglia
Hexb	Microglia
Itgax	Dendritic cells
Cd163	Perivascular macrophages
S100a4	Monocytes and T cells
Rag1	B cells
Trbc2	T cells
Camp	Granulocytes
Cxcl13	Peripheral macrophages
